# Prognostic Model and Nomogram Construction and Validation With an Autophagy-Related Gene Signature in Low-Grade Gliomas

**DOI:** 10.3389/fgene.2022.905751

**Published:** 2022-07-18

**Authors:** Xinrui Li, Zhiyuan Huang, Lei Zhu, Fei Yu, Minghao Feng, Aiqin Gu, Jianxin Jiang, Guangxue Wang, Dongya Huang

**Affiliations:** ^1^ Department of Neurology, Shanghai East Hospital, School of Medicine, Tongji University, Shanghai, China; ^2^ Research Center for Translational Medicine, Shanghai East Hospital, School of Medicine, Tongji University, Shanghai, China; ^3^ Department of Thoracic Surgery, Shanghai East Hospital, School of Medicine, Tongji University, Shanghai, China; ^4^ Department of Neurosurgery, Shanghai East Hospital, School of Medicine, Tongji University, Shanghai, China; ^5^ Department of Neurosurgery, Taizhou People’s Hospital Affiliated to Nanjing Medical University, Taizhou, China

**Keywords:** autophagy, low-grade gliomas (LGG), prognosis, bioinformatics analysis, TCGA TARGET GTEx, HADb

## Abstract

**Background** **:** Autophagy plays a vital role in cancer development. However, the prognostic value of autophagy-related genes (ARGs) in low-grade gliomas (LGG) is unclear. This research aimed to investigate whether ARGs correlated with overall survival (OS) in LGG patients.

**Methods:** RNA-sequencing data were obtained from The Cancer Genome Atlas (TCGA) TARGET GTEx database. Gene Ontology and Kyoto Encyclopedia of Genes and Genomes enrichment analysis of ARGs were performed by the “clusterprofile” R package. Cox regression with the wald χ^2^ test was employed to identify prognostic significant ARGs. Next, the receiver operator characteristic curves were established to evaluate the feasibility of risk score ( $\text{riskscore}=h_{0}\left(\text{t}\right)\text{exp}\left(\overset{n}{\underset{j=1}{\sum }}{\text{Coef}}_{j}\times {\text{X}}_{j}\right)$ ) and other clinical risk factors to predict prognosis. A nomogram was constructed. Correlations between clinical features and ARGs were further verified by a *t*-test or Kruskal–Wallis test. In addition, the correlations between autophagy and immune cells were assessed through the single-sample gene set enrichment analysis (ssGSEA) and tumor immune estimation resource database. Last, the prediction model was verified by LGG data downloaded from the Chinese Glioma Genome Atlas (CGGA) database.

**Results:** Overall, 35 DE-ARGs were identified. Functional enrichment analysis showed that these genes were mainly related to oxidative stress and regulation of autophagy. Nine ARGs (*BAX*, *BIRC5*, *CFLAR*, *DIRAS3*, *GRID2*, *MAPK9*, *MYC*, *PTK6*, and *TP53*) were significantly associated with OS. Age (Hazard ratio (HR) = 1.063, 95% CI: 1.046–1.080), grade (HR = 3.412, 95% CI: 2.164–5.379), histological type (HR = 0.556, 95% CI: 0.346–0.893), and risk score (HR = 1.135, 95% CI: 1.104–1.167) were independent prognostic risk factors (all *p* < 0.05). In addition, *BIRC5*, *CFLAR*, *DIRAS3*, *TP53*, and risk scores were found to correlate significantly with age and tumor grade (all *p* < 0.05). Immune cell enrichment analysis demonstrated that the types of immune cells and their expression levels in the high-risk group were significantly different from those in the low-risk group (all *p* < 0.05). A prognostic nomogram was constructed to predict 1-, 3-, and 5-year survival, and the prognostic value of sorted ARGs were verified in the CGGA database and clinical samples.

**Conclusion:** Our findings suggest that the 9 DE-ARGs’ risk score model could serve as diagnostic and prognostic biomarkers. The prognostic nomograms could be useful for individualized survival prediction and improved treatment strategies.

## Introduction

Based on the 2016 World Health Organization (WHO) Classification of Tumors of the Central Nervous System (2016 CNS WHO), low-grade gliomas (LGG) are a diverse group of primary brain tumors, which include oligodendrogliomas and astrocytomas and are traditionally defined as histological grade 1,2. Also, molecular parameters are used to establish the diagnosis of brain tumors, such as 1p/19q gene deletion and IDH status (Louis et al., 2016). Considering the overlap in clinical and genetic characteristics between IDH wild-type tumors and glioblastoma, the absence of IDH wild-type tumors should be considered low grade (Yan et al., 2009; Eckel-Passow et al., 2015). Researchers estimated that the annual incidence of LGG is approximately 0.7 per 100,000, and that number is still rising now (Rasmussen et al., 2017). It is more likely to occur in healthy people. The main symptom of 80% of LGG patients is a seizure, accompanied by symptoms of focal neurological dysfunction or increased intracranial pressure (Rasmussen et al., 2017). Though patients can benefit from surgical treatment, nearly half of the patients die of recurrence or metastasis after surgery. In recent years, great heterogeneity in prognosis has also been observed (Darlix et al., 2017). Research on the molecular characteristics of gliomas discovered many possible markers including autophagy-related genes (ARGs) for glioma classification, prognosis prediction, and treatment recommendations (Xu et al., 2020; Chen et al., 2021). Therefore, identifying innovative methods and biomarkers is imperative for early detection and new treatment strategies.

Autophagy is a highly conserved cell catabolism process. It is an important mechanism that involves the transport of denatured and aging proteins and the removal of damaged organelles (Ozinsky et al., 2000). Autophagy has recently been reported to be highly associated with tumor incidence, inflammation, therapeutic resistance, and cell death. The above processes are all mediated by ARGs. Previous studies have identified more than 200 ARGs that are directly or indirectly involved in the process of autophagy. At present, growing evidence shows a strong association between autophagy and cancer pathogens. For example, Kong J and Roesly HB et al. reported that autophagy was associated with Barrett’s esophagus and gastroesophageal reflux disease (GERD) (Roesly et al., 2012; Kong et al., 2016). Li C et al. showed that TLR2 promoted the development and progression of human glioma via enhancing autophagy (Li et al., 2019). In addition, ARGs and proteins, including Beclin-1 and p62, have also been studied in esophageal adenocarcinoma (Akashi et al., 2017; Janser et al., 2018). Autophagy may impede glioblastoma cell migration and invasion (Feng et al., 2019). For instance, hyperactive PI3K due to either PTEN mutations or GPR78 overexpression leads to ER stress-mediated cell-lethal autophagy and Caspase3-related apoptosis (Louis et al., 2016; Yi et al., 2020). Likewise, ROS-dependent ERK1/2 signaling leads to glioma cells’ autophagic death via reduction of MMP and GSH/GSSG ratio (Pallichankandy et al., 2015). In addition, studies have shown that some ARGs can be used as prognostic gene signatures of cancer patients and are related to the survival of cancer patients (Hou et al., 2020; Yang et al., 2020; Ren et al., 2021). However, global expression patterns based on autophagy-related gene signatures have not been previously constructed in LGG.

A better understanding of the link between autophagy and LGG, and their relationships with survival in LGG patients, is critical for future LGG diagnosis and therapy. In this study, we aimed to investigate the ARGs’ expression profiles and their values in the prognosis of LGG through bioinformatics analysis. The prognostic value of the sorted ARGs was verified in the Chinese Glioma Genome Atlas (CGGA) database.

## Methods

### Patients’ Samples and Gene Extraction

The RNA-seq data of LGG tissue and normal brain tissue were downloaded from the “TCGA TARGET GTEx” cohort of the UCSC Toil Recompute Compendium (Vivian et al., 2017) (https://xenabrowser.net/datapages/?dataset=TcgaTargetGtex_gene_expected_count). It is a compositive database containing a great quantity of gene expression data derived from healthy people and cancer patients. Transcript expression was quantified using RSEM, and transcriptome alignment had been performed using STAR (GRCh38), using transcripts present in the GENCODE v23 genome annotation. At the transcription level, RSEM expected counts, The Cancer Genome Atlas (TCGA) survival data, and phenotypic data were obtained. (Erady et al., 2021). RSEM expected counts provided by the UCSC Toil Recompute Compendium were log2 (expected_count+1) transformed, and in this analysis, this transformation was removed to produce raw expected counts for us. (Erady et al., 2021). The expected count matrix was used to sort differently expressed ARGs by DESeq2 R package. Clinical data of LGG patients were downloaded from TCGA database (https://portal.gdc.cancer.gov) for prognostic analysis. The FPKM corrected RNA-seq data of TCGA TARGET GTEx was downloaded from UCSC Xena (https://xena.ucsc.edu/) to draw box plots and heatmap drawings. The FPKM corrected RNA-seq data of the TCGA database was used to perform Cox regression. All data processing was performed using Perl software and R software. 232 ARGs were obtained from Human Autophagy Database (HADb, http://www.autophagy.lu), which is a public database providing comprehensive and abundant information about ARGs from PubMed and other biological public databases (Additional File 1: Supplementary Table S1). The expression level of ARGs in the TCGA TARGET GTEx database and TCGA database were extracted through their information in these databases.

### Identification of Differentially Expressed Autophagy-Related Genes

Differentially expressed autophagy-related genes (DE-ARGs) were identified by the DESeq2 R package. The screening criteria were as follows: false discovery rate (FDR) < 0.05, |log_2_ fold change|≥1. To display these DE-ARGs, the heat map, volcano plot, and box plot were drawn by R software.

### Function Enrichment

Functional enrichment analyses of Gene Ontology (GO) and Kyoto Encyclopedia of Genes and Genomes (KEGG) pathway were analyzed on DE-ARGs by the clusterprofile R package. Top results with the FDR < 0.05 were considered to be significant.

### Univariate and Multivariate Cox Proportional Hazard Regression

Univariate Cox proportional hazard regression analysis with the wald χ^2^ test was used to evaluate the correlations between overall survival (OS) of LGG patients from the TCGA database and sorted DE-ARGs. To construct a multivariate Cox regression model, genes with a *p*-value presented by the wald χ^2^ test less than 0.05 were applied. Then, the multivariate Cox regression model was optimized by the AIC value in a stepwise algorithm which worked in the “both” mode of stepwise search. Next, these screened DE-ARGs were applied to calculate risk scores based on their coefficient presented in the multivariate Cox regression model. The risk score was calculated by the following formula: $\text{risk score}=h_{0}\left(\text{t}\right)\text{exp}\left(\overset{n}{\underset{j=1}{\sum }}{\text{Coef}}_{j}\times {\text{X}}_{j}\right)$ , with Coef_j_ representing the coefficient of each DE-ARG, X_j_ representing the relative expression levels of each DE-ARG, and $\;h_{0}\left(\text{t}\right)$ representing the baseline risk function (Tibshirani, 1997). Risk score was applied to construct a prognosis prediction model with other clinical factors by univariate and multivariate Cox regression analysis. A risk score and age were estimated as a continuous variables with one unit change in the Cox regression model. Pathology grade, histology type, and IDH1 mutation were involved in Cox regression analysis as a binary variables.

### Survival Analysis

Patients were divided into high-risk groups and low-risk groups according to their risk scores. The OS of high-risk and low-risk groups was compared by Kaplan–Meier analysis and log-rank test with survival curve. Next, a risk plot, scatter plot, and prognostic heat map were drawn according to the risk score of each patient.

### Clinical Correlation Analysis

Clinical features including age, grade, histology type, and IDH1 mutation were extracted from clinical data downloaded from the TCGA database. The correlations between the prognostic DE-ARGs or risk score and clinical features were analyzed by the *t*-test or Kruskal–Wallis test. The distribution of prognostic DE-ARGs between different categories in each clinical feature was presented by a box plot.

### Receiver Operator Characteristic Analysis

Time-dependent receiver operator characteristic (ROC) curve was drawn to evaluate the sensitivity and specificity of survival prediction of each independent risk factor in distinct years by the survival ROC R package, which was designed for survival analysis with censoring data. The area under the curve (AUC) values range from 0.5 for models without any predictive ability to 1.0 for models with perfect predictive ability.

### Development and Validation of a Nomogram

Independent prognostic factors sorted by Cox regression including risk score and other clinical factors were applied to develop the nomogram. It was validated by C-index and calibration curve to figure out its calibration and discrimination. C-index was calculated with a 100 bootstraps resample.

### Immune Cells and Differentially Expressed Autophagy-Related Genes

In order to explore the relationship between the infiltrating scores of 16 immune cells and the activities of 13 immune-related pathways, we performed the single-sample gene set enrichment analysis (ssGSEA) using the “GSVA” R package (Rooney et al., 2015). The annotated gene set file was provided in Additional file 2: Supplementary Table S2. Moreover, the relationship between immune cells and prognostic genes was collected from the tumor immune estimation resource (TIMER, https://cistrome.shiny apps. io/timer/).

### External Validation of Risk Score and Nomogram

To validate the feasibility of the prognostic model and prognostic prediction value of risk score, the RNA-seq data of LGG tissues with their clinical data were downloaded from the CGGA database (http://www.cgga.org.cn/). The risk score of LGG patients from the CGGA database was calculated according to the formula constructed by LGG samples’ RNA-seq data from the TCGA database and the expression level of prognosis significant DE-ARGs of themselves. Next, patients were divided into high-risk groups and low-risk groups according to risk scores. Kaplan–Meier analysis with log-rank test was applied to figure out the difference in OS rate between the two groups. Univariate Cox regression and multivariate Cox regression were performed to verify the prognosis value of risk score and other available clinical features in the CGGA database. The nomogram was constructed with prognostic predictors including risk score and validated by C-index and calibration curve.

### Experimental Validation

To verify ARG expression levels in LGG and normal brain tissues, we conducted the experimental validation in 15 LGG patients who received surgical tumor resection at the Department of Neurosurgery, Shanghai East Hospital. Ten normal brain tissues were used as a control group. This study was approved by the Internal Review Board of Shanghai East Hospital, Tongji University School of Medicine.

Total RNA was isolated from LGG specimens and normal brain tissues using RNAiso reagent (Takara, Dalian, China) and was reverse-transcribed into first-strand cDNA with a PrimeScript® RT Reagent kit. TB Green® Premix Ex Taq® II kit (Takara) was used to detect the indicated RNA levels on the QuantStudio Real-Time polymerase chain reaction (PCR) System (Applied Biosystem, United States). ACTB was used as an internal control. The primers were synthesized by GENEWIZ company, Suzhou, China. The primers are listed in Additional file 3: Supplementary Table S3.

## Results

### Differentially Expressed Autophagy-Related Genes Between Low-Grade Gliomas and Brain Tissues

**FIGURE 1:**
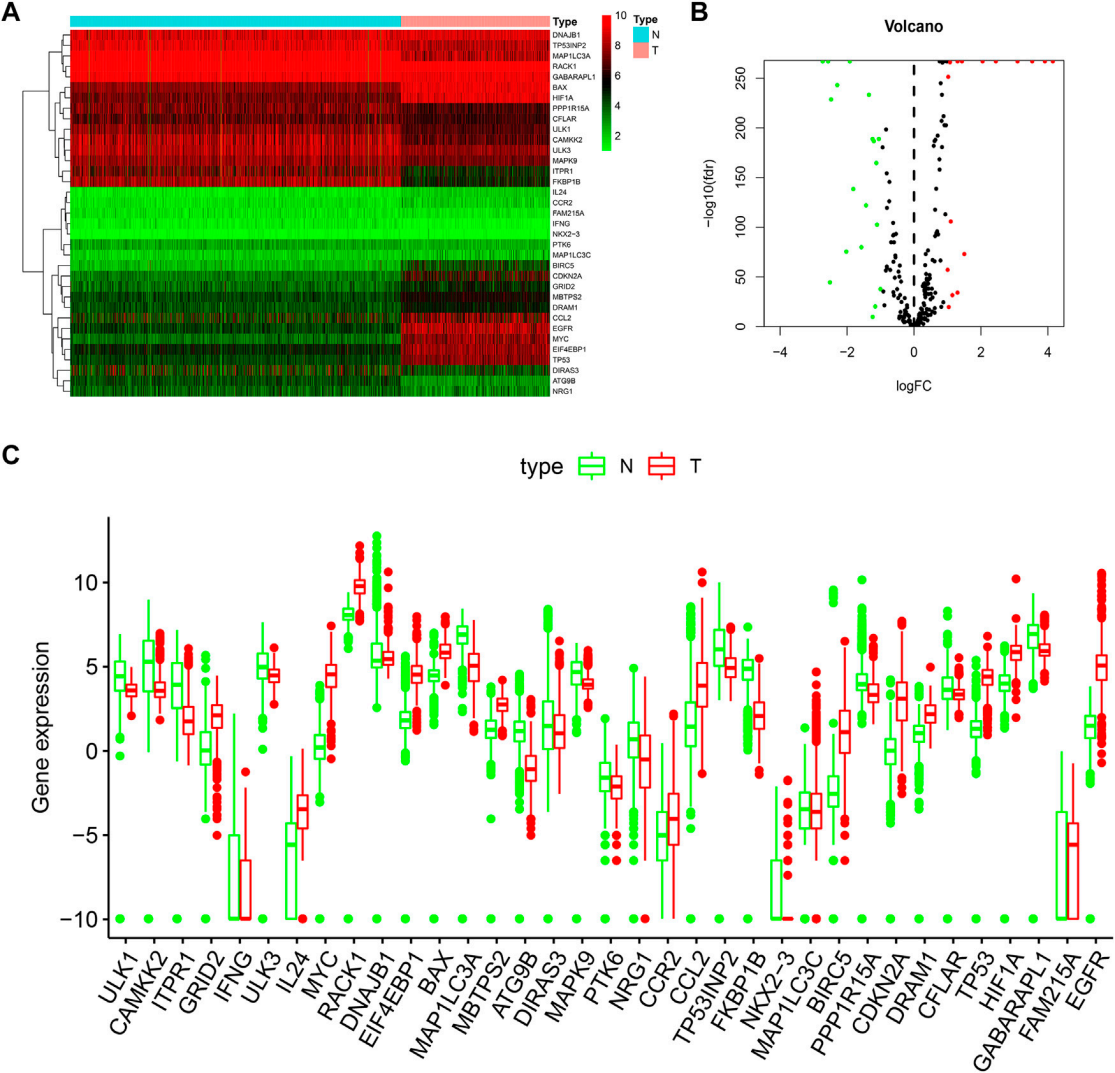
Distributions of DE-ARGs. **(A)** heatmap of DE-ARGs. Green represented down-regulated genes and red represented up-regulated genes. **(B)** volcano plot of SD-ARGs. Green dots represented 19 down-regulated genes; red dots represented 16 up-regulated genes. **(C)** box plot of DE-ARGs in normal brain tissues and tumor brain tissues (all *p* < 0.05).

In this work, we collected mRNA expression profiles and clinical data with 1,148 normal and 520 LGG tissues from the TCGA TARGET GTEx database. (The clinical data including survival time of LGG patient was provided in Additional file 4). Compared with the normal groups, 35 DE-ARGs were found in the LGG groups (Table 1). Among these genes, 19 genes were down-regulated, and 16 genes were up-regulated in the tumor group compared with the normal group. These DE-ARGs were displayed by a heat map, volcano plot, and box plot in Figures 1A–C.

**TABLE 1:** SD-ARGs expression levels in LGG and normal tissue.

Gene	Base mean	logFC	lfcSE	Stat	*p*-Value	FDR
FKBP1B	755.796	−2.728	0.054	−50.596	<0.001	<0.001
ATG9B	199.166	−2.565	0.060	−43.018	<0.001	<0.001
IFNG	1.502	−2.511	0.177	−14.207	<0.001	<0.001
TP53INP2	18,635.505	−2.477	0.076	−32.425	<0.001	<0.001
CAMKK2	7,862.672	−2.293	0.069	−33.441	<0.001	<0.001
DIRAS3	704.514	−2.023	0.109	−18.559	<0.001	<0.001
MAP1LC3A	2,544.961	−1.918	0.044	−43.260	<0.001	<0.001
ITPR1	3,175.201	−1.814	0.072	−25.201	<0.001	<0.001
DNAJB1	9,183.670	−1.572	0.082	−19.102	<0.001	<0.001
PPP1R15A	1996.842	−1.433	0.061	−23.651	<0.001	<0.001
ULK1	3,622.187	−1.347	0.041	−32.760	<0.001	<0.001
CFLAR	2,962.257	−1.239	0.042	−29.457	<0.001	<0.001
NKX2-3	0.433	−1.236	0.191	−6.479	<0.001	<0.001
ULK3	2,836.610	−1.202	0.041	−29.287	<0.001	<0.001
FAM215A	1.190	−1.157	0.122	−9.488	<0.001	<0.001
GABARAPL1	7,391.352	−1.126	0.041	−27.495	<0.001	<0.001
PTK6	40.775	−1.105	0.051	−21.670	<0.001	<0.001
MAPK9	3,558.256	−1.050	0.036	−29.462	<0.001	<0.001
NRG1	152.063	−1.003	0.077	−13.053	<0.001	<0.001
GRID2	501.808	1.004	0.062	16.117	<0.001	<0.001
MBTPS2	620.920	1.022	0.030	34.007	<0.001	<0.001
CCL2	480.185	1.038	0.111	9.358	<0.001	<0.001
BAX	735.789	1.075	0.031	34.999	<0.001	<0.001
DRAM1	226.342	1.098	0.050	22.007	<0.001	<0.001
MAP1LC3C	7.953	1.146	0.096	11.933	<0.001	<0.001
CCR2	7.752	1.300	0.105	12.410	<0.001	<0.001
RACK1	13,287.533	1.301	0.031	41.819	<0.001	<0.001
HIF1A	3,805.729	1.432	0.034	41.646	<0.001	<0.001
IL24	3.365	1.502	0.082	18.248	<0.001	<0.001
EIF4EBP1	298.147	2.048	0.054	37.765	<0.001	<0.001
TP53	720.593	2.445	0.048	51.168	<0.001	<0.001
CDKN2A	132.188	3.093	0.068	45.564	<0.001	<0.001
MYC	644.172	3.532	0.059	60.302	<0.001	<0.001
EGFR	7,014.602	3.910	0.066	59.331	<0.001	<0.001
BIRC5	137.762	4.146	0.079	52.220	<0.001	<0.001

### Function Enrichment Analysis

**FIGURE 2:**
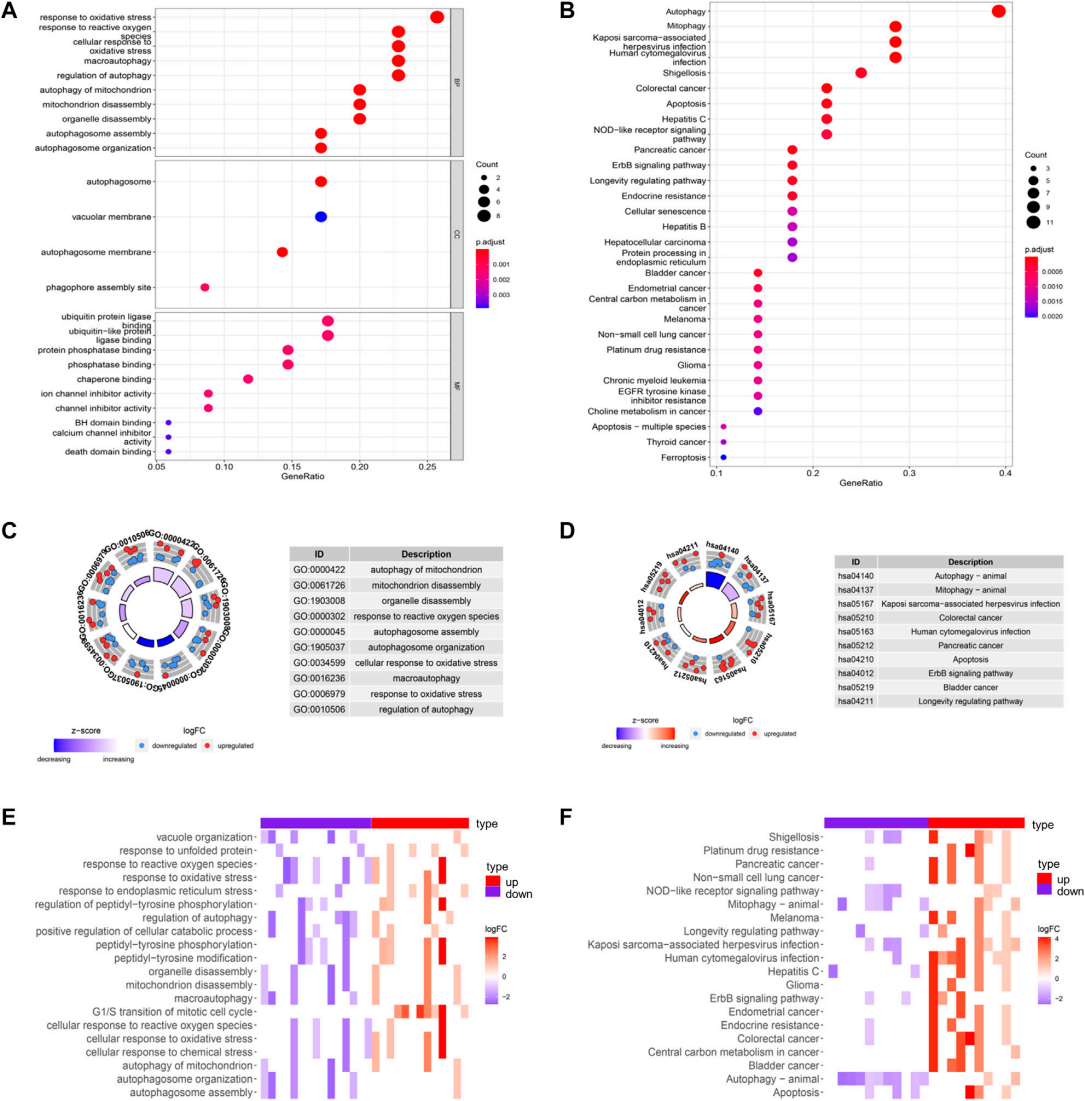
GO and KEGG enrichments of DE-ARGs **(A**,**B)** showed the GO and KEGG enrichment analysis respectively. The larger bubble and darker color indicated the more significant enrichment process. **(C**,**D)** enrichment pathways in the GO and KEGG circle plots, respectively. The inner-circle indicated Z-score. The red color represented the significant enrichment. The outer circle indicated the various pathways, in which the blue dots indicated down-regulated genes, and the red was up-regulated genes. **(E**,**F)** heat maps of GO and KEGG enrichment, respectively. The red color represented the up-regulated genes, and purple represented the down-regulated genes.

Biological process (BP), cellular component (CC), and molecular function (MF) categories are important components of GO analysis. Figure 2A showed the GO functional enrichment analysis. In the aspect of BP, DE-ARGs were mostly enriched in the response to oxidative stress and regulation of autophagy. In the aspect of CC, DE-ARGs were mainly enriched in the autophagosome process. In the MF, DE-ARGs were mostly enriched in ubiquitin-protein ligase binding and ubiquitin-like protein ligase binding process. Besides, KEGG analysis showed that DE-ARGs were mainly enriched in autophagy (Figure 2B).

GO circle plot showed that these DE-ARGs were mainly enriched in autophagy of mitochondrion. The circle plots of KEGG pathways clearly showed that these genes were mainly involved in autophagy. Many DE-ARGs exhibit other pathways related to autophagy, such as mitophagy and apoptosis shown in the circle plots (Figures 2C,D). Corresponding heatmaps for GO and KEGG revealed that DE-ARGs were enriched in the response to the oxidative stress process and the development of some cancers (Figures 2E,F).

### Prognosis-Related Differentially Expressed Autophagy-Related Genes

**FIGURE 3:**
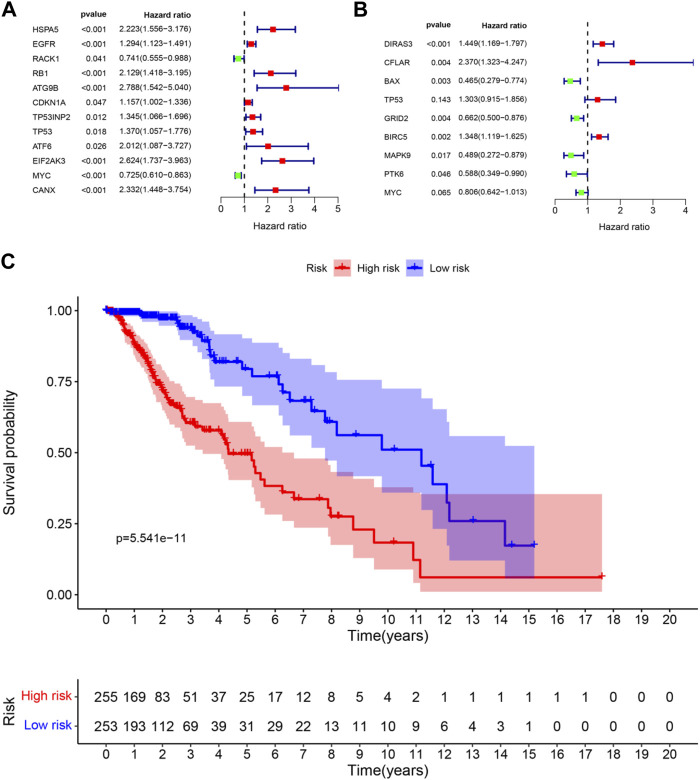
Forest plots and Kaplan–Meier curve **(A)** forest plot of univariate Cox regression for 12 prognosis-related DE-ARGs; **(B)** forest plot of multivariate Cox regression for 9 sorted prognosis-related DE-ARGs; **(C)** Kaplan–Meier curve for LGG patients’ OS in the high-risk and low-risk groups when stratified by the autophagy-related risk score (FDR correction had been used for differential genetic screening, and *p*-values in Cox regression were provided by the wald χ^2^ test, and no further FDR correction is required.)

Univariate regression analysis was used to eliminate genes that were not associated with the prognosis of LGG. 12 ARGs were found to be significantly associated with OS of LGG patients (shown in Figure 3A). 9 ARGs (*DIRAS3, CFLAR, BAX, TP53, GRID2, BIRC5, MAPK9, PTK6, MYC*) were selected (shown in Table 2 and Figure 3B) by multivariate Cox regression after model optimizing by AIC value. Among these nine genes, *DIRAS3, CFLAR*, *TP53*, and *BIRC5* played risk roles in the survival of LGG patients (HR > 1), while the other five genes (*BAX*, *GRID2*, *MAPK9*, *PTK6*, and *MYC*) were potential protective factors for LGG patients (HR < 1). The risk score of each patient was calculated according to the expression level and coefficient of these prognosis significant ARGs presented in the multivariate Cox regression model. 10 patients’ data in the TCGA database were waived because of incomplete survival data or mismatch between RNA-seq data and survival data. Next, the median risk score value was applied as a cutoff point for classifying the LGG patients into a high-risk group (*n* = 255) and a low-risk group (*n* = 253), respectively. The number of cases differed between the two groups due to the lack of survival time of two patients in the low-risk group. Patients in the high-risk group were accompanied by lower OS than patients in the low-risk group (median time = 4.34 vs. 11.19 years, *p* < 0.001, Figure 3C).

**TABLE 2:** ARGs associated with prognosis.

Gene name	Coefficient	HR	95% CI	P-value
DIRAS3	0.371	1.449	1.169-1.797	0.001
CFLAR	0.863	2.370	1.323-4.247	0.004
BAX	−0.766	0.465	0.279-0.774	0.003
TP53	0.265	1.303	0.915-1.856	0.143
GRID2	−0.413	0.662	0.500-0.876	0.004
BIRC5	0.299	1.348	1.119-1.625	0.002
MAPK9	−0.715	0.489	0.272-0.879	0.017
PTK6	−0.532	0.588	0.349-0.990	0.046
MYC	−0.215	0.806	0.642-1.013	0.065

Nine ARGs were related with OS and used to calculate the risk score to classify the tumor patients into high and low risk groups. $\text{Risk score}=h_{0}\left(\text{t}\right)\text{exp}\left(\overset{n}{\underset{j=1}{\sum }}{\text{Coef}}_{j}\times {\text{X}}_{j}\right)$ , $\;h_{0}\left(\text{t}\right)$ : baseline risk function, exp.: ARGs expression level, n: quantity of significant DE-ARGs, Coefj: coefficient of each DE-ARG, Xj: relative expression levels of each DE-ARG.

### Performance of Risk Signature in Low-Grade Gliomas From The Cancer Genome Atlas

**FIGURE 4:**
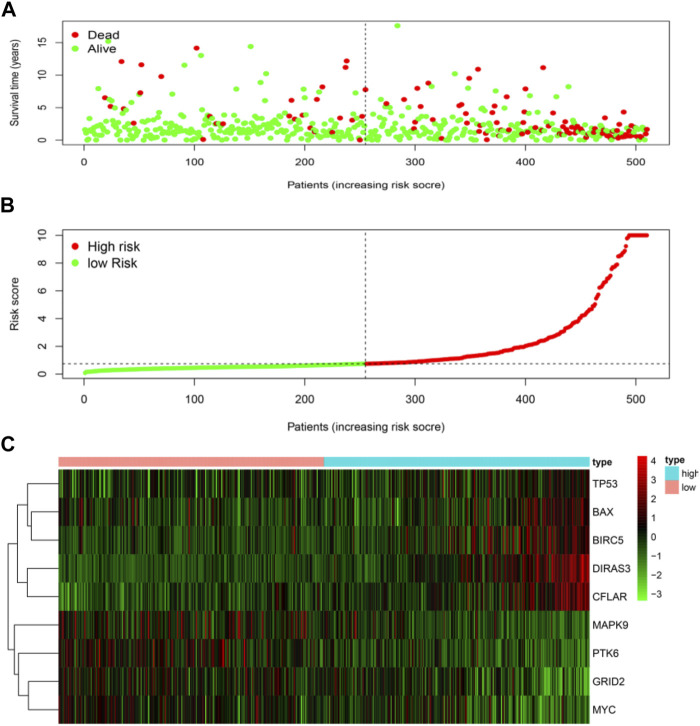
Risk score analyses of high and low-risk groups in tumor patients. **(A)** risk score scatters plot of high risk and low risk. Red dots represented the dead patients and green represented the alive. With the increase in risk scores, more patients died. **(B)** dotted line indicates the individual inflection point of the risk score curve, by which the patients were categorized into low-risk and high-risk groups. LGG patients were presented as red point (high-risk) and green point (low-risk). **(C)** risk score heatmap of nine ARGs. The colors from green to red indicate the expression level of genes varies from low to high.

The risk score was calculated for each patient who suffered from LGG. The patient whose risk score was higher than the median of all the patients was defined as a high-risk group. On the contrary, it was defined as a low-risk group. Patients with higher risk scores were expected to demonstrate increased risks of death and poor survival outcomes (Figures 4A,B). The risk heat map showed *PTK6*, *GRID2*, *MAPK9*, and *MYC* were up-regulated in the low-risk group, and *DIRAS3*, *CFLAR*, *BAX*, *TP53*, and *BIRC5* were up-regulated in the high-risk group (Figure 4C).

### Independent Prognostic Prediction Factors of Overall Survival

**FIGURE 5:**
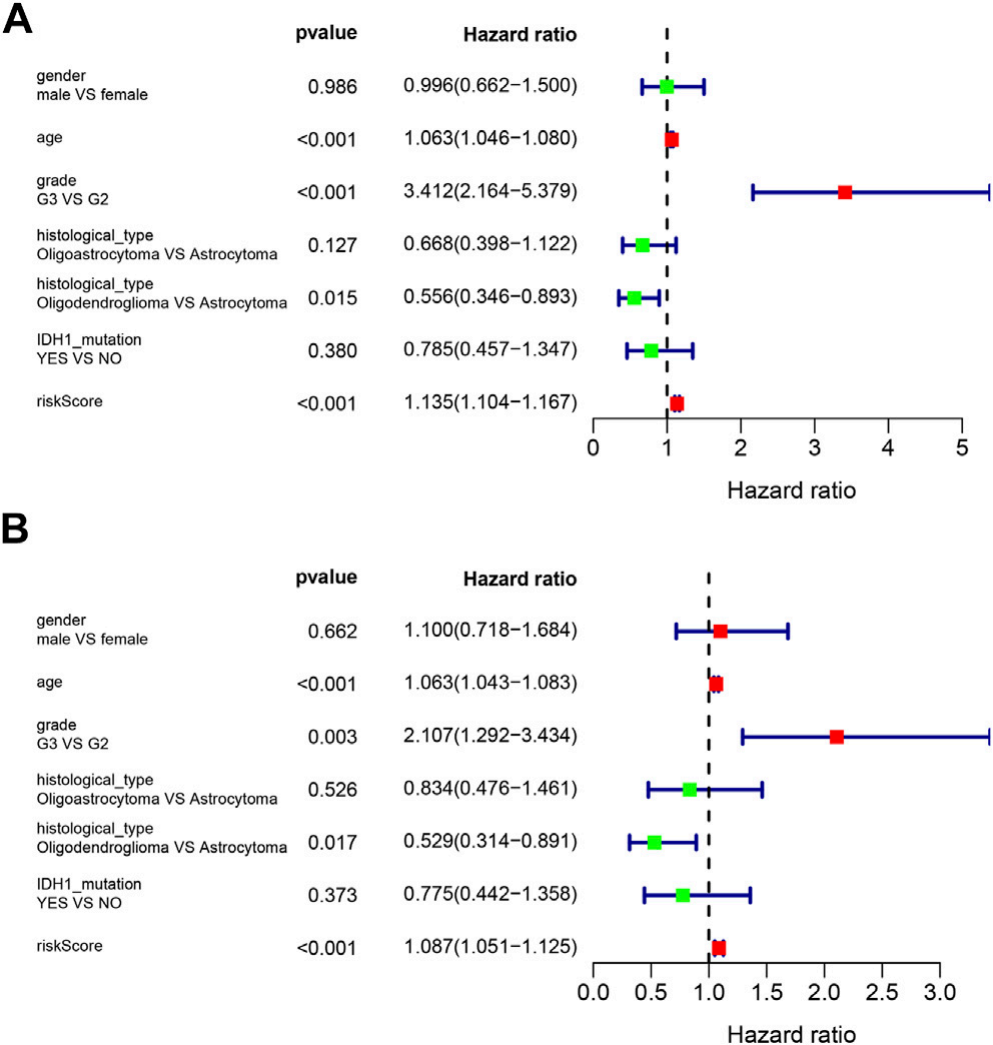
Forest plots of prognostic risk factors **(A)** univariate Cox regression forest plot. **(B)** multivariate Cox regression forest plot of independent risk factors.

Univariate Cox and multivariate Cox regression were used to analyze the clinical features and the risk score for association with OS. Variables with a *p*-value less than 0.05 in the wald χ^2^ test in Cox regression were recognized as independent prognostic factors for OS of LGG patients. The univariate Cox regression analysis showed that age (HR = 1.063, 95% CI: 1.046–1.080), grade (G3 vs. G2, HR = 3.412, 95% CI: 2.164–5.379), histological type (Oligodendroglioma vs. Astrocytoma, HR = 0.556, 95% CI: 0.346–0.893), and risk score (HR = 1.135, 95% CI: 1.104–1.167) were significantly correlated with OS (all *p* < 0.05) (Figure 5A). Multivariate Cox regression showed that age (HR = 1.063, 95% CI: 1.043–1.083), grade (G3 vs. G2, HR = 2.107, 95% CI: 1.292–3.434), histological type (Oligodendroglioma vs. Astrocytoma, HR = 0.529, 95% CI: 0.314–0.891), and risk score (HR = 1.087, 95% CI: 1.051–1.125) were independent risk factors for survival (all *p* < 0.05) (Figure 5B).

### Clinical Correlation Analysis

**FIGURE 6:**
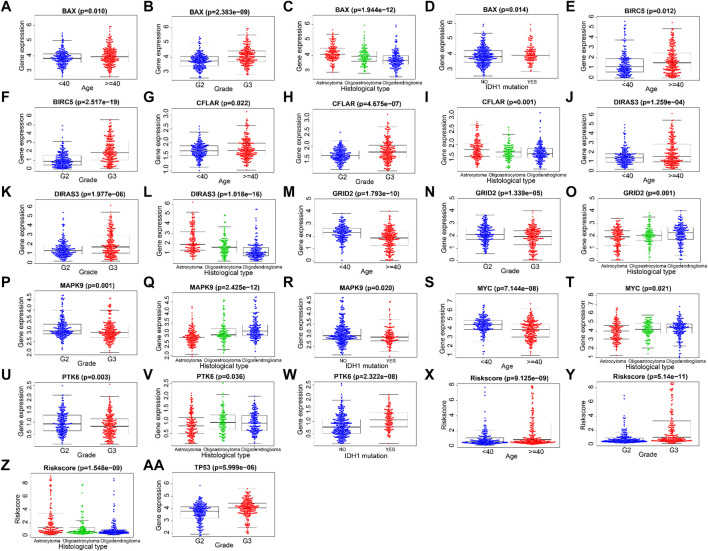
Correlations between ARGs and clinical features.

The correlations between the prognosis-related ARGs and clinical features were verified by the *t*-test or Kruskal–Wallis test based on the number of categories of each clinical feature. Next, the results showed that the expression level of *GRID2* decreased with the increased age and/or pathological grade. Also, a similar conclusion can be drawn in the correlation analysis of *PTK6*, *MAPK9*, and *MYC*. On the contrary, the expression level of *BAX*, *BIRC5*, *CFLAR*, *DIRAS3*, and *TP53* showed upward trends with the increase of age and/or pathological grade, which might indicate their diverse roles compared with *GRID2*, *PTK6*, *MAPK9*, and *MYC* in the development of LGG. Risk scores also increased with the growth of age and ascending of pathological grade. The expression level of *BAX*, *MAPK9*, and *PTK6* was distinct in different IDH1 mutation statuses. This information might be helpful for the development of the targeted drugs. *BAX*, *CFLAR*, *DIRAS3*, *GRID2*, *MAPK9*, *MYC* genes, and risk scores were different in distinct pathological types, which revealed the clue of the potential pathogenetic mechanism of each pathological type respectively (all *p* < 0.05) (Figure 6A-AA, Table 3).

**TABLE 3:** Clinical correlation analysis.

Gene Name	Gender (*p*-value)	Age (*p*-value)	Grade (*p*-value)	Histological Type (*p*-value)	IDH1 Mutation (*p*-value)
DIRAS3	1.876 (0.061)	−3.873 (<0.001)	−4.83 (<0.001)	73.647 (<0.001)	0.66 (0.510)
CFLAR	0.257 (0.798)	−2.307 (0.022)	−5.131 (<0.001)	13.416 (0.001)	0.885 (0.377)
BAX	−0.12 (0.905)	−2.576 (0.010)	−6.106 (<0.001)	53.933 (<0.001)	−2.481 (0.014)
TP53	0.795 (0.427)	−1.951 (0.052)	−4.586 (<0.001)	0.548 (0.760)	−0.978 (0.329)
GRID2	0.698 (0.485)	6.542 (<0.001)	4.407 (<0.001)	13.685 (0.001)	1.464 (0.145)
BIRC5	0.718 (0.473)	−2.534 (0.012)	−9.517 (<0.001)	5.582 (0.061)	1.027 (0.305)
MAPK9	0.848 (0.397)	0.797 (0.426)	3.282 (0.001)	53.49 (<0.001)	2.348 (0.020)
PTK6	0.589 (0.556)	1.633 (0.103)	3.009 (0.003)	6.672 (0.036)	−5.777 (<0.001)
MYC	−1.012 (0.312)	5.487 (<0.001)	0.154 (0.878)	7.733 (0.021)	−0.284 (0.777)
Risk score	1.103 (0.271)	−5.936 (<0.001)	−6.891 (<0.001)	40.573 (<0.001)	1.265 (0.207)

### Development and Validation of Prediction Model

**FIGURE 7:**
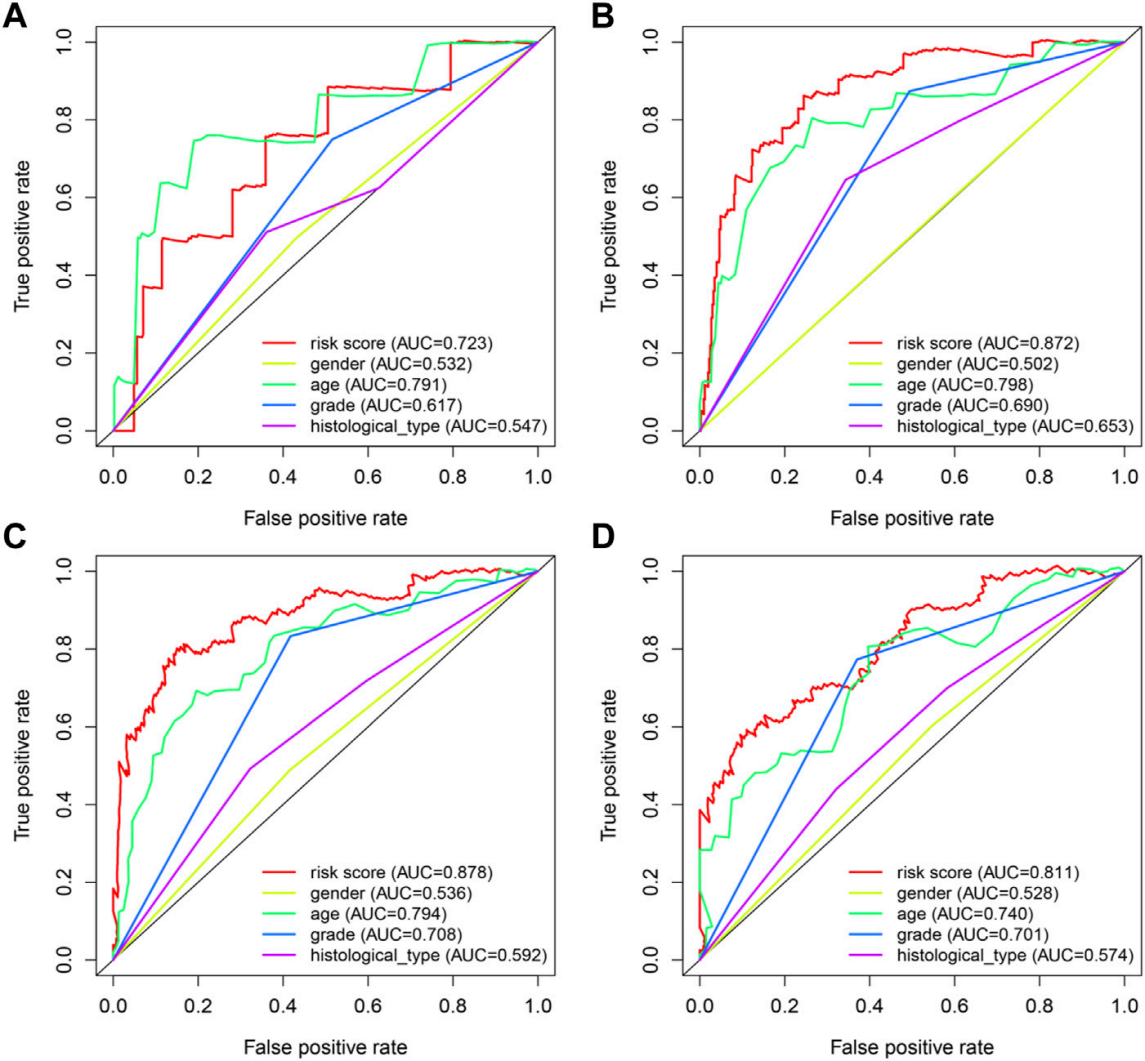
ROC curves of predicting survival. **(A)** 0.5-year ROC curve **(B)** 1-year ROC curve **(C)** 3-year ROC curve **(D)** 5-year ROC curve AUC: area under the curve. The larger AUC is, the more accurate it predicts.

**FIGURE 8:**
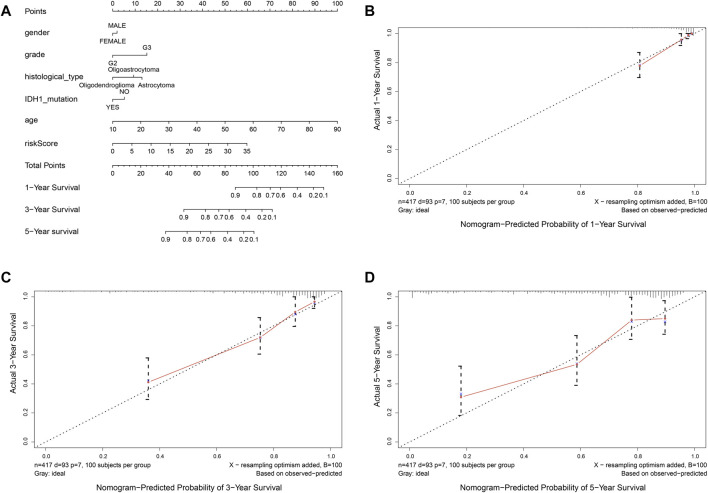
Nomogram to predict the overall survival of patients who suffer from LGG. **(A)** nomogram to predict 1-, 3-, or 5-year OS. **(B-D)** calibration curve for nomogram to predict 1-, 3-, or 5-year OS. The *x*-axis is nomogram-predicted survival, and the *y*-axis is actual survival. The reference line is 45^◦^ inclined and indicates perfect calibration.

In order to provide an approach to predicting the survival, we constructed the 0.5-year, 1-year, 3-year, and 5-year ROC curves using the independent risk factors associated with OS (age, grade, histological type, and risk score), respectively. In addition, the prediction feasibility of each independent risk factor was assessed by the AUC. According to the ROC curve, the risk score showed a better ability to predict the 1–5 years’ survival (1-year AUC = 0.872; 3-year AUC = 0.878; 5-year AUC = 0.811) than other indicators, while the age showed better ability to predict the 0.5 year’s survival (AUC = 0.791) (Figures 7A–D). Nomograms for OS prediction in LGG patients were created with independent prognostic factors including age, grade, histological type, and risk score. (Figure 8A). The C-index of this nomogram is 8.373, which showed satisfactory discrimination. In addition, the calibration curves for the nomogram showed a favorable calibration ability of this nomogram in both 1-, 3-, and 5-year. (Figures 8B–D).

### Immune Cells Enrichment Analysis

**FIGURE 9:**
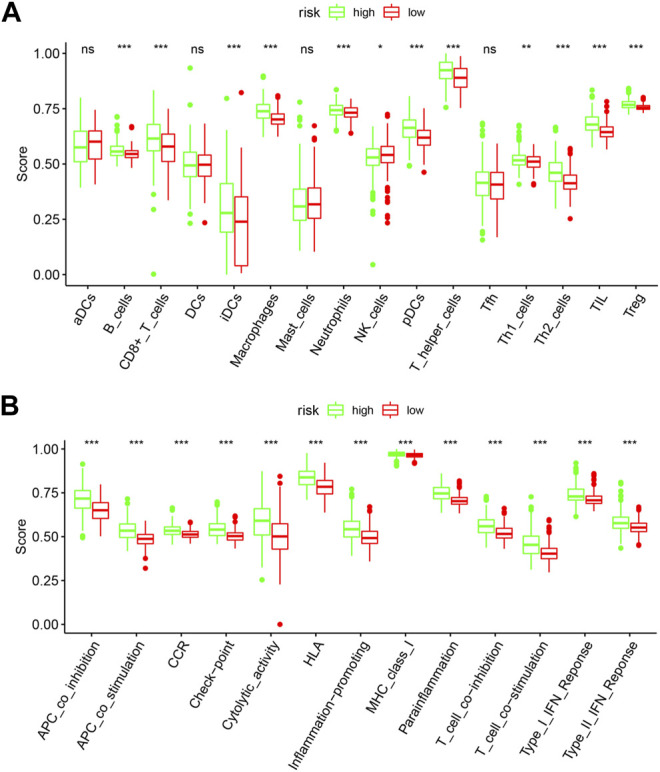
Comparison of the ssGSEA scores between the high-risk and low-risk groups. The score of 16 immune cells **(A)** and 13 immune-related functions **(B)** are displayed in boxplots. DCs: dendritic cells; iDCs: immature DCs; pDCs: plasmacytoid dendritic cells; TIL: tumor-infiltrating lymphocyte; CCR: cytokine-cytokine receptor; APC: antigen-presenting cells. Adjusted *p*-values were shown as the following: ns, not significant; *p < 0.05; **p < 0.01; ***p < 0.001.

To further explore the relationships between the risk scores and immune cells and functions, we quantified the enrichment scores of 16 immune cell subpopulations and their related functions with the ssGSEA R package. The results showed that the types of immune cells (such as B cells, CD8^+^ T cells, iDCs, macrophages, neutrophils, NK cells, pDCs, T helper cells, Th1 cells, Th2 cells, TIL, and Treg) in the high-risk group were significantly different with those in the low-risk group (Figure 9A). Moreover, the scores of all listed immune functions in Figure 9B were significantly higher in a high-risk group, implying their immunological functions associated with autophagy were more active in the high-risk group.

**FIGURE 10:**
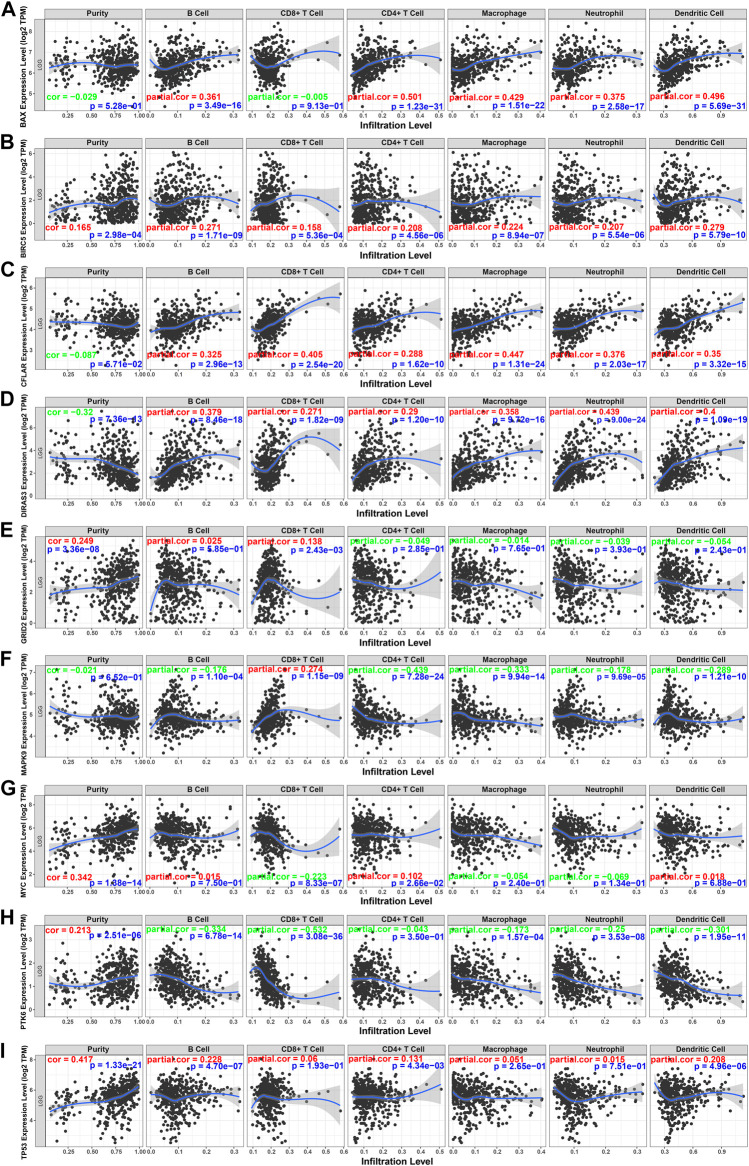
Relations between immune cells and prognostic genes. **(A)** BAX expression level and immune cells in low-grade glioma; **(B)** BIRC5 and immune cells; **(C)** CFLAR and immune cells; **(D)** DIRAS3 and immune cells; **(E)** GRID2 and immune cells; **(F)** MAPK9 and immune cells; **(G)** MYC and immune cells; **(H)** PTK6 and immune cells; **(I)** TP53. TPM: transcripts per kilobase million. The red color in the correlation coefficient represents a positive correlation, and the green color represents a negative correlation.

To better understand the characteristics of immune cells and their relations with ARGs, the TIMER database was used to analyze the correlation between the abundance of immune cells and the nine prognostic genes (*BAX, BIRC5, CFLAR, DIRAS3, GRID2, MAPK9, MYC, PTK6,* and *TP53*). *BAX, CFLAR*, and *DIRAS3* were positively correlated with B cells, CD4 + T cells, macrophages, neutrophils, and dendritic cells (Figures 10A,C,D), but *PTK6* was negatively correlated with these immune cells (Figure 10H). Likewise, *BIRC5* and *TP53* were positively correlated with all immune cells (Figures 10B,I). Positive correlations were found between *GRID2, MYC* expression, and the infiltration of B cells (Figures 10E,G) and negative correlations between *MAPK9* expression and the infiltration of B cells, CD4 + T cells, macrophages, neutrophils, and dendritic cells (Figure 10F).

### External Validation of Risk Score and Nomogram

**FIGURE 11:**
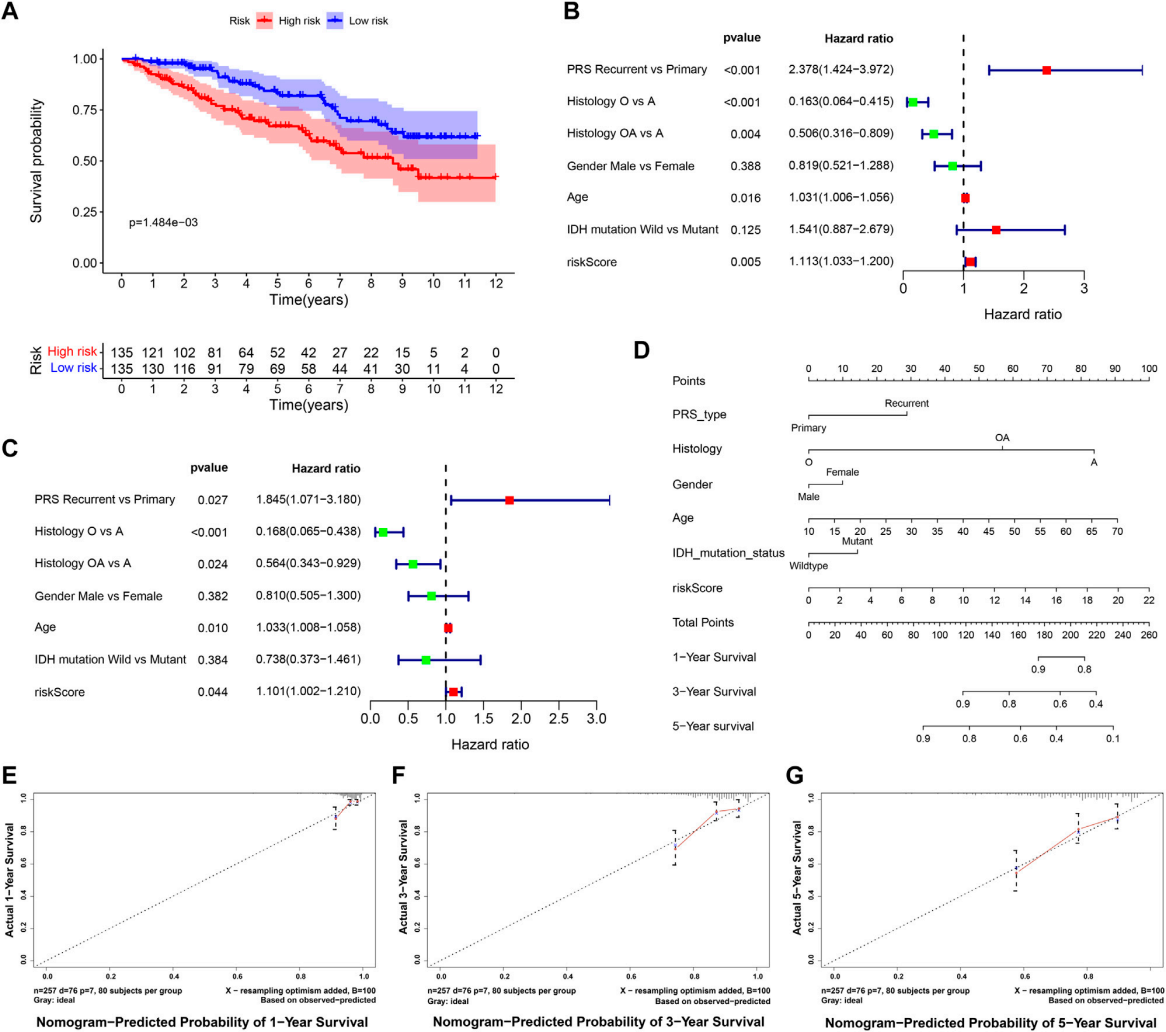
Validation of risk score calculated by prognosis significant DE-ARGs **(A)** Kaplan–Meier analysis of patients from the high-risk group and low-risk group. **(B,C)** forest plot of univariate and multivariate Cox regression for prognosis indicators including risk score (histology types: A: Astrocytoma, O: Oligodendroglioma OA: Oligoastrocytoma). **(D,G)** nomogram with calibration curve in 1-year, 3-year, and 5-year.

The RNA-seq transcriptome data and clinical information from the CGGA database were verified to determine whether the 9 DE-ARGs demonstrated similar prognostic values in different populations. Results showed that the OS rate was lower in the high-risk group (Figure 11A). Univariate and multivariate Cox regression analysis showed that histology type, age, and risk score were significantly correlated with the OS of LGG patients, which were consistent with the result drawn by data from the TCGA TARGET GTEx database (Figures 11B,C). Nomogram constructed by risk score and other clinical features was validated by C-index and calibration curve (Figure 11D). The C-index was 7.388, and the calibration curves were presented in Figures 11E–G. According to the validation performed by data from the CGGA database, the risk score calculated by our sorted significant prognosis DE-ARGs can be recognized as an independent prognosis prediction factor.

### Experimental Validation

**FIGURE 12:**
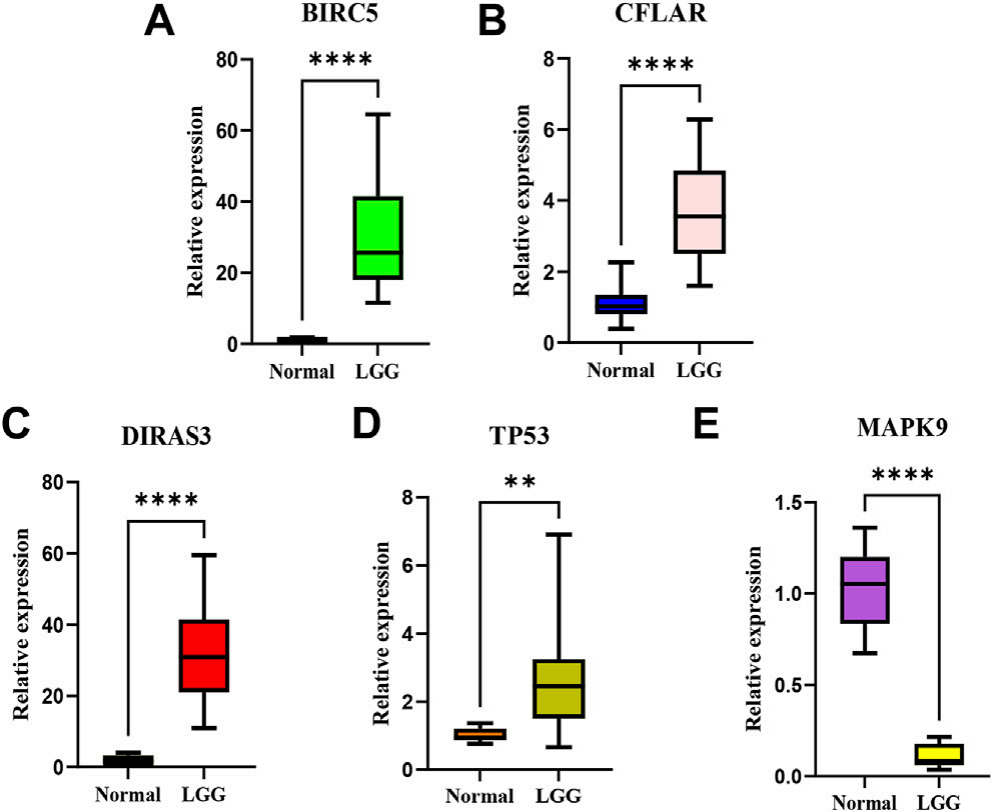
The relative expression levels of the five genes in normal brain tissues and LGG tissues.The *BIRC5* **(A)**, *CFLAR* **(B)**, *DIRAS3* **(C),** and *TP53* **(D)** were up-regulated, and *MAPK9* **(E)** was significantly down-regulated in LGG tissues. **p < 0.01; ****p < 0.0001.

According to the screening and validation steps described above, we selected the five most significant ARGs (*BIRC5, CFLAR, DIRAS3, TP53, MAPK9*) from the 35 significantly different genes, according to the *p* (<0.000001) and FDR values (<0.000001), and ACTB was set as an internal reference. By analysis, quantitative real-time PCR (qRT-PCR) results showed the *BIRC5, CFLAR, DIRAS3,* and *TP53* were up-regulated, and *MAPK9* was significantly down-regulated in LGG tissues (*p* < 0.001). The details of the five genes were visualized in Figure 12 A–E (Additional file 5 and 6).

## Discussion

Autophagy is one of the metabolic processes for eukaryotic cells to maintain cellular homeostasis by eliminating damaged organelles and proteins via autophagosomes (Mao et al., 2021). Emerging evidence demonstrated that autophagy plays a crucial role in the development of cancers (White et al., 2015; Amaravadi et al., 2019; Li et al., 2020). At the early stage of tumor progression, autophagy acts as a tumor suppressor, and in advanced stages, autophagy promotes cancer survival (Kondo et al., 2005). The effect of autophagy on cells is a “double-edged sword” because if autophagy is maintained at a high level, it will lead to autophagy death. We performed a bioinformatic analysis using the available data on LGG focused on the differentially expressed genes of the autophagy process. Then, functional enrichment analysis was performed on DE-ARGs. It may help explore the mechanism of origination of LGG with autophagy, such as the signaling pathways activated during the development of LGG or cell organelles involved in LGG development. Besides, a nomogram was constructed to facilitate the verification and employment in clinical practice.

In this study, we first identified 35 DE-ARGs based on the TCGA TARGET GTEx database. Functional enrichment analysis showed that these DE-ARGs were mainly enriched in GO and KEGG pathways related to oxidative stress, regulation of autophagy, and process utilizing autophagic mechanism, providing strong evidence that autophagy plays a significant role in the development of LGG. 9 ARGs, including *DIRAS3, CFLAR, BAX, TP53, GRID2, BIRC5, MAPK9, PTK6,* and *MYC*, and significant correlations with the prognosis were found by univariate and multivariate Cox regression analyses. *BIRC5, CFLAR, DIRAS3,* and *TP53* played a risk role in the survival of LGG patients, which were verified in the risk score heatmap. Researchers suggested that the high expression levels of these four genes may be related to poor prognosis.

Several studies strongly supported the associations between these DE-ARGs and cancers. *BIRC5* (also named survivin) is a well-known cancer therapeutic target. Shuhei Suzuki et al. showed that survivin inhibitors could sensitize glioma stem cells to osimertinib by reducing survivin expression to prevent migration, proliferation, and metastasis from gliomas (Suzuki et al., 2019). This research indirectly proved that BIRC5 was a risk factor for LGG. *BIRC5* interacts with Beclin1 to regulate the lipid kinase Vps-34 protein and promote the formation of Beclin1-Vps34-Vps15 core complexes, thereby inducing autophagy. (Kang et al., 2011). Many researchers in other directions also drew consistent conclusions with us. CFLAR, encoded by the *CFLAR* (Caspase 8 and FADD-like apoptosis regulator) gene, is a regulator protein that induces apoptosis and is structurally similar to caspase-8. Wang. J et al. reported that in positive regulation of the I-κB kinase/NF-κB signaling pathway (target gene *CFLAR*), *CFLAR* plays a critical role in autophagy, necroptosis, and apoptosis (Wang J. et al., 2017). In particular, higher CFLAR expression has been associated with inferior survival in one acute myeloid leukemia cohort 50 and chemotherapy resistance in several tumor types51-53 (Supper et al., 2021). Previous reports showed that the various isoforms of *CFLAR* can control the threshold of autophagy when overexpressed in cell lines. Another study by Simone Fulda revealed that *CFLAR* participated in many cellular pathways like autophagic cell death (Galluzzi et al., 2017). For therapeutic purposes, targeting *CFLAR* might be a feasible strategy. *DIRAS3* (DIRAS family, GTP-binding RAS-like 3) encodes a member of the ras superfamily. The encoded protein, DIRAS3, plays role in autophagy in certain cancer cells by regulating the autophagosome initiation complex. Nutrient deprivation can cause transcriptional upregulation of *DIRAS3*-mediated autophagy (Sutton et al., 2019). Next, overexpression of *DIRAS3* promotes LGG cell proliferation and invasion via the EGFR-AKT signaling pathway (Wang et al., 2020). Another study showed that *DIRAS3* was overexpressed in LGG and was positively associated with adverse outcomes in LGG patients (Wang et al., 2020). Therefore, silencing the expression of *DIRAS3* may demonstrate an inhibitory effect on LGG metastasis accompanied by a long-lasting tumor suppression effect theoretically. *TP53* (tumor protein p53) responds to diverse cellular stresses to regulate the expression of target genes, thereby inducing cell cycle arrest, apoptosis, autophagy, senescence, DNA repair, or changes in metabolism (Yin et al., 2002). Mutations in this gene are associated with a variety of human cancers, including LGG (Marcel et al., 2010). The TP53 mutation is one of the most frequent genetic alterations in LGG (Ohgaki and Kleihues, 2007), and several studies reported associations between *TP53* polymorphisms and LGG risk (Stacey et al., 2011; Shi et al., 2012). Smita Bhatia et al. (Wang X. et al., 2017) identified an association between SNP rs2909430 on the *TP53* gene and LGG risk. The R280T mutation in *TP53* has been reported in human glioma and is involved in promoting cell proliferation (Lin et al., 2012). The above-mentioned studies are consistent with our results.

Aside from autophagy, many other functions of ARGs have been found and studied by the GO and KEGG pathways, which include response to oxidative stress, apoptosis, ubiquitin-like protein ligase binding process, and some infections processes. Researchers documented that autophagy could affect reactive oxygen species and oxidative stress response, thus regulating the biological properties of some metabolic factors (Ma et al., 2018). Oxidative stress can lead to apoptosis through numerous mechanisms, and apoptosis has been considered one of the most important mechanisms for cell death. Several lines of evidence suggest that autophagy may promote cell apoptosis, and inhibition of the autophagy could decrease apoptosis (Maiuri et al., 2007; Luo et al., 2019). Next, the intricate details between autophagy and apoptosis trigger pivotal crosstalk in tumor suppression (Macintosh and Ryan, 2013). The ubiquitin signal is thought to be the autophagy-lysosome pathway’s target. Ubiquitin signals are essential during autophagy to selectively integrate proteins, organelles, and microbial intruders into autophagosomes (Grumati and Dikic, 2018). Two ubiquitin-like binding systems, particularly the combination of ATG12 and ATG5, and the conversion of LC3 into a membrane-bound form of phosphatidylethanolamine binding are involved in autophagosome formation. (Mizushima, 2007). The interplay of extracellular signals and autophagy is the most recent development of research in proteoglycan signaling (Schaaf et al., 2019). Proteoglycan promotes tumor cell migration and survival, as well as the formation of blood vessels, by activating or inhibiting angiogenesis and autophagy in tumor parenchyma and surrounding stromal cells (Iozzo, 2005; Iozzo and Sanderson, 2011; Wight, 2018). Christine Z et al. reported that autophagy operated as an antiviral process during human cytomegalovirus (HCMV) infection, thus demonstrating a protective effect to the organism. (Zimmermann et al., 2021). Overall, these prognostic ARGs may contribute to early detection and may be an effective strategy for increasing survival chances.

When LGG patients were divided into two groups according to the risk score calculated by the nine ARGs’ signatures, a remarkable diversity can be seen in the Kaplan–Meier survival curve between the high-risk and low-risk patients. Also, we evaluated the relations between ARGs and patients’ clinical features. The results showed the expression level of *BIRC5, CFLAR,* and *DIRAS3,* and *TP53* showed upward trends with the tumor stage and grade increased, which indicated that ARGs were involved in the progression of LGG. Further investigation found that risk score was also one of the independent prognostic factors via multivariate Cox regression analysis, which suggested that the autophagy gene could serve as an accurate survival indicator. Risk scores could distinguish between high-risk and low-risk patients for guiding individualized treatment. Furthermore, time-dependent ROC showed that risk score demonstrated a relatively higher prognostic accuracy in predicting survival of LGG patients in the first, third, and fifth years than other clinical features in our study, implying its great potential as a new class of biomarkers in cancer. Whereas, clinical concerns about age only demonstrated a relatively higher prognostic accuracy in the first half-year. The reason for the relatively high accuracy of the age factor in predicting only 0.5 years of survival in LGG patients may be related to the treatment modality of LGG patients. Research showed that radiotherapy, chemotherapy, and surgery were used for LGG patients, and younger patients demonstrated better tolerance to these treatments (Delgado-López et al., 2017; Nunna et al., 2021). Nomograms, as a predictive tool, provide an individualized risk score for a given patient, which facilitates the development of a more precise treatment strategy (Iasonos et al., 2008). In recent years, nomogram has become increasingly popular due to its ability to use different variables to construct statistical prediction models (Brockman et al., 2015; Ma et al., 2019; Cui et al., 2020). The calibration curve showed that the predicted value of the nomogram was in good agreement with the real value. Our model can provide a new orientation for prognostic risk assessment and individualized treatment strategy selection for LGG patients.

The immune microenvironment of cancer cells plays an instrumental role in tumor development (Deng et al., 2019). Our results demonstrated that the immune status was significantly different between the low-risk and high-risk LGG patients. In addition, the scores of all listed immune functions, including APC coinhibition, cytolytic activity, T cell coinhibition, and type I IFN response et al., were significantly higher in the high-risk group, indicating the complexity between autophagy and immunity.

Further analysis and external validation of our study showed that risk score was an independent risk factor of prognosis, which suggested that autophagy genes could serve as an accurate survival indicator. At last, a smaller cohort of 15 clinical samples (LGG tissues) and 10 normal brain tissues were collected to verify the above findings. Results showed that *BIRC5, CFLAR, DIRAS3,* and *TP53* were up-regulated, and *MAPK9* was significantly down-regulated in LGG tissues (*p* < 0.001), which were in agreement with the model predictions.

The strength of our study is that we performed a systematic analysis of autophagic genes from the national database, which provided robust statistical support. It was different from previous research (Qu et al., 2020; Chen et al., 2021) in that we succeeded in not only identifying the autophagy-related gene signature in LGG patients but also validating the results by external validation and RT-qPCR data obtained from clinical samples. Also, double validation made our conclusions more reliable. This study still exhibits some limitations. First, a small number of LGG samples and limited clinical information in the TCGA database limited the accuracy of the prognosis predictive model. Detailed information about neuroimaging and treatment methods was not recorded in the nomogram. Second, the prediction model needs further validation in multicenter and large-scale clinical trials. Third, the molecular mechanism of autophagy affecting the prognosis of LGG patients and its significance for clinical translational therapy need to be further studied. Notwithstanding its limitations, this study provides a comprehensive overview of the ARGs profile in LGG. These issues may be addressed if a larger study is to be conducted.

## Conclusion

In conclusion, a risk prediction model based on *BAX, BIRC5, CFLAR, DIRAS3, GRID2, MAPK9, MYC, PTK6,* and *TP53* was constructed, which could predict the prognosis of LGG patients and provide therapeutic targets for clinical treatment. The prognostic nomogram offers the possibility for individualized survival prediction and improvement of treatment strategies. These biomarkers could be further applied to clinical assessments to validate our findings.

## Data Availability

The datasets presented in this study can be found in online repositories. The names of the repository/repositories and accession number(s) can be found in the article/Supplementary Material.

## Abbreviations

ARGs, Autophagy-related genes; AUC, Area under the curve; BAX, BCL2-associated X protein; BIRC5, Baculoviral IAP repeat-containing protein 5; BP, Biological process; CC, Cellular component; CFLAR, Caspase 8 and FADD-like apoptosis regulator; CGGA, Chinese Glioma Genome Atlas; DE-ARGs, Differentially expressed autophagy-related genes; DIRAS3, DIRAS family, GTP-binding RAS-like 3; ER, Endoplasmic reticulum; FDR, False discovery rate; GSH/GSSH, Glutathione/ Glutathione (Oxidized); GO, Gene Ontology; GRID2, Glutamate receptor, ionotropic, delta 2; HADb, Human Autophagy Database; KEGG, Kyoto Encyclopedia of Genes and Genomes; LGG, Low-grade gliomas; MAPK9, Mitogen-activated protein kinase 9; MF, Molecular function; MMP, Mitochondrial membrane potential; MYC, V-myc myelocytomatosis viral oncogene homolog (avian); OS, Overall survival; PTK6, PTK6 protein tyrosine kinase 6; ROC, Receiver Operating Characteristic; TCGA, The Cancer Genome Atlas; TP53, Tumor protein p53.
