# Supplementary material for: Prognostic Model and Nomogram Construction and Validation With an Autophagy-Related Gene Signature in Low-Grade Gliomas
Source: Front Genet. 2022 Jul 18;13:905751. doi: 10.3389/fgene.2022.905751 (PMC9342864; doi:10.3389/fgene.2022.905751)
Supplement: Supplementary file 3 [file Table6.DOCX]

**Supplementary Table S1 Primers design and their sequences of ARGs**

| **Number** | **Gene Primer** | **Base sequence(5'to 3')** | **Base number** | **Purification method** |
| --- | --- | --- | --- | --- |
| 1 | BIRC5-F1 | TGACGACCCCATAGAGGAAC | 20 | tPAGE |
| 2 | BIRC5-R1 | CGCACTTTCTCCGCAGTTT | 19 | tPAGE |
| 3 | CFLAR-F1 | CTGTCCTTGTTCCTCGTCCC | 20 | tPAGE |
| 4 | CFLAR-R1 | AGTGGGGGAGTTGCCCG | 17 | tPAGE |
| 5 | DIRAS3-F1 | GTGCCTCCGAGAAAGGGGTC | 20 | tPAGE |
| 6 | DIRAS3-R1 | CCAAAGCTGGCGTTACCCAT | 20 | tPAGE |
| 7 | MAPK9-F1 | CCCAAGGGATTGTTTGTGCTG | 21 | tPAGE |
| 8 | MAPK9-R1 | GCTCTCTTTGCATGAGTTTGGT | 22 | tPAGE |
| 9 | TP53-F1 | CTTCCCTGGATTGGCAGC | 18 | tPAGE |
| 10 | TP53-R1 | TTTCAGGAAGTAGTTTCCATAGGT | 24 | tPAGE |
| 11 | ACTB-F1 | TGACAGACTACCTCATGAAGATCC | 24 | tPAGE |
| 12 | ACTB-R1 | CTGCTTGCTGATCCACATCTG | 21 | tPAGE |
