# Supplementary material for: Prognostic Model and Nomogram Construction and Validation With an Autophagy-Related Gene Signature in Low-Grade Gliomas
Source: Front Genet. 2022 Jul 18;13:905751. doi: 10.3389/fgene.2022.905751 (PMC9342864; doi:10.3389/fgene.2022.905751)
Supplement: Supplementary file 7 [file Table10.DOCX]

Dear editor:

Thank you very much for your careful review and kind work with regard to our manuscript “**Prognostic model and nomogram** **construction and validation with an autophagy-related gene signature in low-grade gliomas”**. We have studied the valuable comments from the last Frontiers journal carefully and tried our best to revise the manuscript as follows according to the nice comments.

1. Clarified the definition of LGG and WHO classification.
2. Corrected the mistakes and ambiguities in the figures.
3. Validated the results by external validation and TR-qPCR data obtained from clinical samples (LGG tissues and normal brain tissues).
4. According to ROC curve, the risk score showed a better ability to predict the 1 to 5 years’ survival (1 year AUC= 0.872; 3 years AUC=0.878; 5 years AUC=0.811) than other indicators (Figure 7 A-D). Gender, age, grade and histological type are all the clinically common markers. Therefore, we have reason to believe that risk score had higher predictive ability and clinical application value than other indicators in LGG.
5. We quantified the enrichment scores of 16 immune cell subpopulations and their related functions with the ssGSEA R package to further explore the relationships between the risk scores and immune cells and functions. To better understand characteristics of immune cells and their relations with ARGs, the TIMER database was used to analyze the correlation between the abundance of immune cells and the nine prognostic genes. These data can provide some scientific basis for the further study of LGG.

We have submitted our manuscript with tracked changes to highlight the revisions.

We appreciate for editors’ warm work earnestly, and hope that the corrections will meet with approval. Please feel free to contact us with any questions and we are looking forward to your consideration.

With best regards,

Dongya Huang

Mar 28, 2022
